# Stewart B. Palmer, M.D.S.

**Published:** 1903-05

**Authors:** 


### STEWART B. PALMER, M.D.S.

Under the proper head will be found a full and appreciative article upon the life and character of Dr. Palmer, from the pen of a correspondent very closely connected with him professionally in past years.

It is perhaps too early to analyze a character such as Dr. Palmer possessed. The personal influence he had over men during his life possibly has a tendency to warp judgment, not only in his case, but of all who have lived worthily and died honorably.

While this is true, it is difficult to feel that Dr. Palmer's worth as a man and as a dentist can be overstated. He had, like all original men, his peculiarities. These very frequently led to misconceptions of his true character. His scientific papers were not always understood and oftentimes not appreciated by the unthinking, but to the few they contained golden grains of pure thought, indicating deeper views in a substratum of invisibility.

Dr. Palmer's part in what was known as the "New Departure" did more, probably, to make for him a national character than his original work. This curious chapter in the dental history of this country may never be written out, but to those who were actively engaged in professional labor at the time, it presents, in memory, a vivid picture, in which Drs. Palmer, Flagg, and Chase are prominent figures, and back in the shadow stand the amazed fifteen thousand dentists of the country. They felt that the dictum of the Fathers, "That what was worth filling at all was worth filling with gold," was being trampled in the dust by sacrilegious hands. For a time this trinity of iconoclasts stood the severe criticism of the entire profession, the general feeling being that their views would never become part of the dental professional creed.

The faith in gold as a filling-material was, however, shaken. The articles written by this trinity of thought made eventually an impression, and caused men to stop and ask themselves, "Am I not doing myself and my patients an injury by this devotion to one material and one method in filling teeth?" Years have passed since this question was first asked and the answer may be read to-day in a modified practice in which all things available are regarded as proper for use in the salvation of teeth. The heat of that controversy has died, but, while we must regard the exponents as extremists, it must be conceded that great innovations and great reforms are only made possible by radical utterances and radical practice. The subservient individual, the "begging-your-pardon" man never stirs the world. Dr. Palmer was not one of these. Truth to him was of peculiar worth, not to be bartered for upon the counters of good opinion of the world. He sought it where it is best found, in communion with nature, and when these original investigations seemed to evolve facts to his comprehension, no power, however antagonistic, could change his position. Hence the flood of criticism of the new departure passed over him and left him unharmed. His gentle but uncompromising spirit said,

"Wait, time will show whether that which we believe be true or false."

This period in Dr. Palmer's life may be the most interesting to the biographer, but while it is of great value, the writer feels that it is not the most important or most impressive. His skill in his profession was recognized long before this time. He had much to do with the training of young men in a private way, and the result of his influence has been marked in the history of some of the most advanced in the dental profession. The love that these bear their preceptor is really the best monument to his worth in the world.

Dr. Palmer has been gathered into the realms of historical remembrance. He will be classed with that body of earnest men who made dentistry a possibility in this country. The doors have opened and shut many times to allow these to pass on to their eternal work in other spheres of activity, but of all those who have entered therein and passed from view, none will appeal more to loving remembrance than this gentle but indomitable spirit who showed his love for men and his profession best by being true to his highest conception of truth.

